# Comparison of quality of life after stereotactic body radiotherapy and surgery for early-stage prostate cancer

**DOI:** 10.1186/1748-717X-7-194

**Published:** 2012-11-20

**Authors:** Alan Katz, Montserrat Ferrer, José Francisco Suárez

**Affiliations:** 1 Flushing Radiation Oncology, 4022 Main St # 3, Flushing, NY, 11354, USA; 2 Health Services Research Unit, IMIM (Hospital del Mar Research Institute), Barcelona, Spain; 3 CIBER en Epidemiología y Salud Pública (CIBERESP), Barcelona, Spain; 4 Universitat Autònoma de Barcelona, Bellaterra, Spain; 5 Servicio de Urología, Hospital Universitari de Bellvitge, L’Hospitalet de Llobregat, Barcelona, Spain

**Keywords:** Prostate cancer, Prostatectomy, Quality of life, Stereotactic body radiotherapy

## Abstract

**Background:**

As the long-term efficacy of stereotactic body radiation therapy (SBRT) becomes established and other prostate cancer treatment approaches are refined and improved, examination of quality of life (QOL) following prostate cancer treatment is critical in driving both patient and clinical treatment decisions. We present the first study to compare QOL after SBRT and radical prostatectomy, with QOL assessed at approximately the same times pre- and post-treatment and using the same validated QOL instrument.

**Methods:**

Patients with clinically localized prostate cancer were treated with either radical prostatectomy (n = 123 Spanish patients) or SBRT (n = 216 American patients). QOL was assessed using the Expanded Prostate Cancer Index Composite (EPIC) grouped into urinary, sexual, and bowel domains. For comparison purposes, SBRT EPIC data at baseline, 3 weeks, 5, 11, 24, and 36 months were compared to surgery data at baseline, 1, 6, 12, 24, and 36 months. Differences in patient characteristics between the two groups were assessed using Chi-squared tests for categorical variables and t-tests for continuous variables. Generalized estimating equation (GEE) models were constructed for each EPIC scale to account for correlation among repeated measures and used to assess the effect of treatment on QOL.

**Results:**

The largest differences in QOL occurred in the first 1–6 months after treatment, with larger declines following surgery in urinary and sexual QOL as compared to SBRT, and a larger decline in bowel QOL following SBRT as compared to surgery. Long-term urinary and sexual QOL declines remained clinically significantly lower for surgery patients but not for SBRT patients.

**Conclusions:**

Overall, these results may have implications for patient and physician clinical decision making which are often influenced by QOL. These differences in sexual, urinary and bowel QOL should be closely considered in selecting the right treatment, especially in evaluating the value of non-invasive treatments, such as SBRT.

## Background

The early-stage prostate cancer treatment landscape is crowded with effective surgical and non-surgical treatment options, yet, no randomized controlled trials to date have proven the superiority of any one treatment approach in terms of cancer control [1,2]. Technical advances have added additional treatment options such as stereotactic body radiation therapy (SBRT) for which several recent studies have shown promising biochemical control [3-6]. Given this breadth of treatment options the treatment decision process has become complex, particularly for low-risk prostate cancer patients where the prognosis is likely to be favorable independent of which treatment they choose. Indeed, foregoing immediate treatment and initiating active surveillance is an increasingly attractive option for many patients [7]. Wilt et al. [8] recently found in a randomized study that neither all-cause nor prostate-cancer mortality were significantly different in low-risk patients undergoing prostatectomy or active surveillance, although prostatectomy may reduce mortality in patients with higher PSA scores or intermediate- and high-risk cancer. In this setting, then, factors such as the invasiveness and length of treatment, treatment-related toxicity, and the impact of treatment on quality of life (QOL), play increasingly important roles in the treatment decision process.

The impact of treatment on prostate cancer patients’ QOL has been the focus of a variety of longitudinal studies [9-14]. The instrument has been extensively validated [15] and applied successfully in Spanish-speaking patients [9,16]. Most recently, Pardo et al. published a longitudinal, comparative QOL study using the validated Expanded Prostate Cancer Index Composite (EPIC) QOL instrument for patients receiving surgery, external-beam radiation therapy or brachytherapy but who did not receive hormonal therapy [16]. Consistent with other findings, this study demonstrated radical prostatectomy was associated with urinary incontinence and sexual dysfunction whereas the radiation treatments were associated with short-term decrements in bowel QOL and gradual sexual QOL declines.

SBRT is a rapid, non-invasive treatment that precisely delivers radiation to the moving prostate while limiting radiation to surrounding normal tissues thereby offering the potential for minimal toxicities while maintaining patient QOL [17]. Whereas traditional radiation therapy imposes a 8–9 week treatment duration, SBRT has a one week treatment duration that is comparable to radical prostatectomy, but without being invasive. SBRT efficacy may also be comparable to radical prostatectomy; recent 5-year biochemical disease-free survival rates of 93% have been reported [3]. Given the absence of any QOL comparison to date between radical prostatectomy and SBRT, we sought to compare QOL for SBRT and radical prostatectomy.

## Methods

### Study design and participants

This was a retrospective study of clinically localized prostate cancer patients treated with either radical prostatectomy or SBRT. Radical prostatectomy patients were participants in the Spanish Multicentric Study of Clinically Localized Prostate Cancer from April 2003 to March 2005. Details of the study are described elsewhere [9]. Briefly, the patients had stage T1 or T2 prostate cancer, no previous transurethral prostate resection, and were treated at one of 10 Spanish hospitals. For the purpose of this analysis, only patients who received surgery and who did not receive neoadjuvant or adjuvant hormonal therapy were included. The patients underwent radical retropubic prostatectomy with nerve-sparing at the surgeon’s discretion. Research protocols were approved by the ethics review boards of each hospital and informed written consent was obtained for all patients.

SBRT patients were selected from a larger group of 304 patients treated at Winthrop University Hospital (Mineola, NY) from April 2006 to July 2008. The patients included in the present study were only those treated at least 3 years prior to analysis and not treated with hormones. In addition, 31 patients from the larger group were lost to follow-up or were dead of other causes and were not included here. All patients signed consent statements and were informed of the potential risks involved with this treatment. Institutional IRB-approval was obtained on the treatment protocol. Details of the treatment are described elsewhere [5]. Briefly, SBRT was delivered to patients with clinical stage T1c or T2b prostate cancer using the CyberKnife (Accuray Inc., Sunnyvale, CA). Inverse treatment planning was performed using a CT scan (1.5-mm cuts), with MRI fusion where feasible two weeks after fiducial placement. All pretreatment imaging was performed with the patient in the same position used for treatment delivery. Changes in the position of the prostate during treatment were tracked and automatically corrected for with the aid of gold fiducial markers implanted within the prostate. The first 38 patients received a total dose of 35 Gy delivered in 5 daily fractions; the remaining patients received a total dose of 36.25 Gy in 5 daily fractions. The dose was prescribed to a planning target volume (PTV) created by a 5-mm expansion of the prostate gross tumor volume (GTV), with a 3-mm posterior expansion. For intermediate- and high-risk patients, the seminal vesicles were included in the GTV. For high-risk patients, an 8-mm margin was added on the involved side. All patients had the bladder, prostate, rectum, seminal vesicles and penile bulb contoured, but the urethra was not identified. On each treatment day patients received 1500 mg of amifostine (MedImmune, LLC Gaithersburg, MD) mixed in saline instilled into the rectum [18,19]. As in the prostatectomy group, patients receiving hormonal therapy were excluded.

### Quality of life measure

At baseline, patient’s age, T stage, prostate-specific antigen (PSA) level, Gleason histological grading scores, prostate volume, and QOL were assessed. Patient risk was assigned using the D’Amico et al. [1] definition of risk. For the surgery patients, QOL assessment was administered centrally by telephone interviews before and 1, 3, 6, 12, 24, and 36 months after treatment. For the SBRT patients, QOL assessment was administered in person at baseline and either in person or by telephone at 3 weeks, 5, 11, 24, and 36 months. QOL was assessed using the Expanded Prostate Cancer Index Composite (EPIC) [15]. The EPIC instrument (50 items) was constructed by expanding the University of California-Los Angeles Prostate Cancer Index to assess function and bother in the four domains (urinary, bowel, sexual, and hormonal). All EPIC items are answered on a 5-point Likert scale. For each domain a summary score were constructed as recommended by the developers of the questionnaire. EPIC scores range from 0 to 100 with higher scores reflecting better QOL. For comparison purposes, SBRT EPIC data at 3 weeks, 5, 11, 24, and 36 months were compared to surgery data at 1, 6, 12, 24, and 36 months. The Spanish version of the EPIC administered to the surgery patients has shown equivalence with the original version [20].

### Statistical analysis

Differences in patient characteristics between the two patient populations were assessed using Chi-squared tests for categorical variables and t-tests for continuous variables. Differences in baseline QOL scores were assessed using t-tests. Statistical significance was established at α = 0.05, and changes in QOL that exceeded half a standard deviation from the baseline value were defined as clinically significant [21]. Generalized estimating equation (GEE) models were constructed for each EPIC scale to account for correlation among repeated measures and used to assess the effect of treatment on QOL. The GEE models included baseline patient age as a continuous variable, risk group as a categorical variable, and prostate volume as a continuous variable as adjusting factors. Variables included in the model were chosen because their clinical significance is clear in practice and in the literature on prostate cancer treatment, and they were statistically significant in the bivariate analyses. Time was included as a categorical variable in the model using the baseline, 1, 6, 12, 24 and 36 month time points. Surgery was used as the reference group in these GEE models. GEE allows the presence of missing values in the repeated measurements of the dependent variable, without having to exclude individuals with incomplete data and with no need of imputation methods (even though missing completely at random does not hold) [22]. The GEE statistical analyses were carried out using SAS 9.3 software, all other analyses were carried out using GraphPad Prism 5.04.

## Results

A total of 216 SBRT patients and 123 surgery patients were included in this analysis. As summarized in Table 1, the patient characteristics were significantly different between treatment groups for age, baseline PSA, Gleason score, T Stage, risk and prostate volume. Overall the SBRT patients were older, with lower baseline PSA values. In addition, more of the SBRT patients had low-risk disease and their prostate volumes were larger. Furthermore, the use of medications for erectile function differed between the two patient populations. A small percentage of the SBRT patients retained sexual function with the use of medications such as Sildenafil whereas fewer of the surgery patients used such aids.

**Table 1:** Patient characteristics for the SBRT and surgery cohorts

**Patient centeracteristics**	**SBRT**	**Surgery**	p*
**Number of Patients**	216	123	
**Age** (**years**)			< 0.0001
Median	69.25	64.91	
min	43.83	44.76	
max	89.29	74.82	
Age Group			< 0.0001
< 60	34 (15.7%)	25 (20.3%)	
60–69	88 (40.7%)	77 (62.6%)	
>70	94 (43.5%)	21 (17.1%)	
**PSA** (**ng**/**ml**)			< 0.0001
mean	6.13	7.96	
median	5.37	7.40	
min	0.74	3.80	
max	20.50	22.60	
group			< 0.0001
< 4	32 (14.8%)	2 (1.6%)	
4–10	165 (76.4%)	94 (76.4%)	
> 10	19 (%)	27 (22.0%)	
**Gleason**			*p* = 0.0050
<7	162 (75.0%)	71 (57.7%)	
7	52 (24.1%)	48 (39.0%)	
>7	2 (0.9%)	3 (2.4%)	
unknown	0 (0.0%)	1 (0.8%)	
**T Stage**			< 0.0001
T1	191 (88.4%)	82 (66.7%)	
T2	25 (11.6%)	41 (33.3%)	
**Risk**			< 0.0001
Low	156 (72.2%)	52 (42.3%)	
Intermediate	56 (25.9%)	67 (54.5%)	
High	4 (1.9%)	4 (3.3%)	
**Prostate Volume** (**cc**)†			*p* = 0.005
median	57.30	52.41	
min	16.8	12.0	
max	224.0	152.0	

†The prostate volume was unknown for 20 surgery patients.

Table 2 compares baseline EPIC QOL between the two patient populations. Baseline EPIC urinary QOL was significantly different with the SBRT patients having lower baseline urinary QOL. Sexual QOL was significantly different between the SBRT patients and all surgery patients; SBRT patients had higher sexual QOL at baseline. Comparison of baseline sexual QOL between the SBRT patients and the patients for whom a nerve-sparing surgical procedure was used, however, showed no significant difference. Baseline bowel QOL was comparable between the two treatment groups.

**Table 2:** Baseline EPIC Quality of life scores

**Quality of life scores**	**SBRT**	**Surgery**	p*
**Urinary**			< 0.0001
Mean	89.27	95.25	
SD	8.34	12.27	
**Sexual**			0.0400
Mean	57.78	52.57	
SD	22.34	22.29	
		**Nerve**-**Sparing**	0.6920
Mean	57.78	59.41	
SD	22.34	16.30	
		**Non**-**Nerve**-**Sparing**	0.0079
Mean	57.78	50.17	
SD	22.34	23.65	
**Bowel**			0.1441
Mean	95.47	96.43	
SD	6.05	5.33	

**Figure 1:**
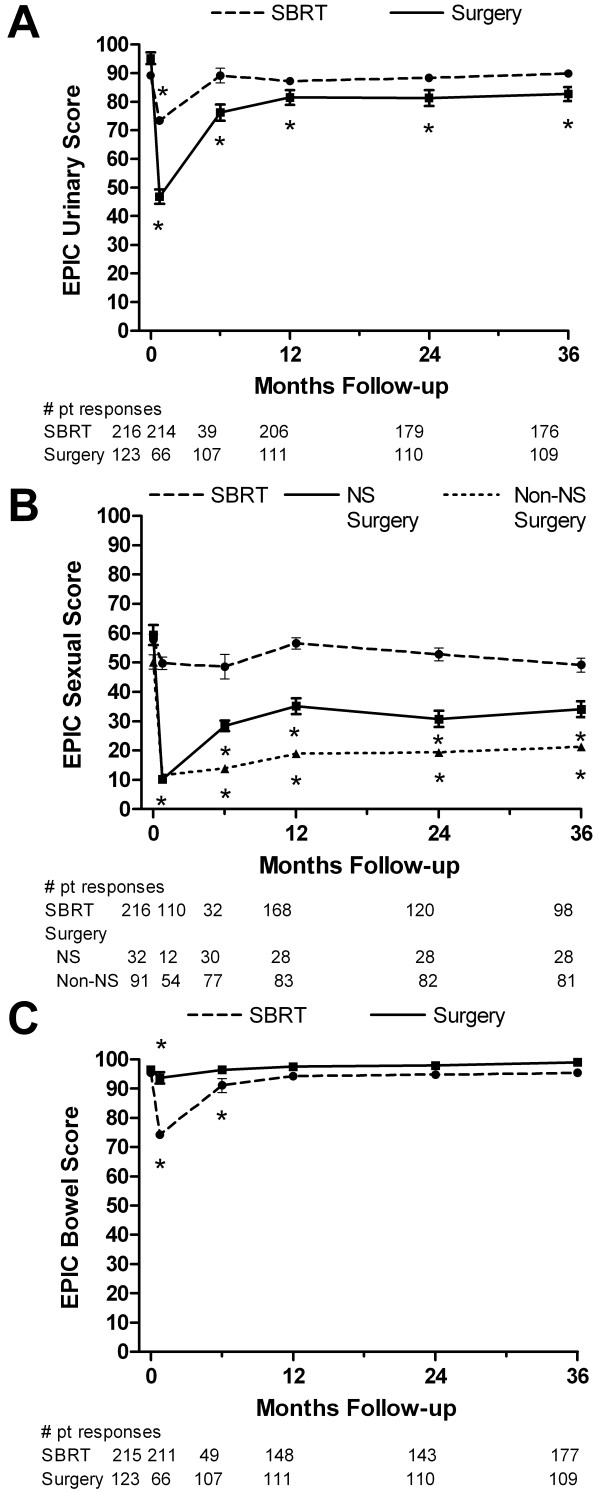
**Unadjusted mean EPIC quality of life scores plotted over time for (A) urinary, (B) sexual, and (C) bowel quality of life.** The SBRT patient scores are shown with a dashed line and the surgery patients are shown with a solid line. EPIC domain scores range from 0 to 100 with higher values indicating higher quality of life. For the sexual domain, the surgery scores are divided into those patients receiving the nerve-sparing (solid line) and non-nerve-sparing (dotted line) techniques. Asterisks (*) denote time points at which scores were clinically significantly different from baseline. Error bars denote 95% confidence intervals.

Mean EPIC QOL scores over time for the SBRT and surgery patients are shown in Figure 1. Mean urinary scores for surgery patients exhibited a clinically significant decline at 1 month-post treatment, improved at 6 and 12 months, but remained clinically significantly lower than at baseline at all follow-up times. Mean urinary scores for SBRT patients also exhibited a clinically significant decline at 1 month post-treatment. The mean urinary score for SBRT patients improved by 6 months and was no longer clinically significantly different at all subsequent follow-up times.

Given the differences in baseline EPIC QOL scores for surgery patients receiving nerve-sparing and non-nerve-sparing procedures the EPIC surgery QOL scores were examined as two separate groups: nerve-sparing and non-nerve-sparing. Independent of surgical approach, all surgery patients experienced a clinically significant decline in EPIC sexual QOL at 1 month. At 6 months, patients receiving non-nerve-sparing surgery retained clinically significantly lower sexual QOL with only modest improvements by 36 months. Patients receiving nerve-sparing surgery also retained clinically significantly lower sexual QOL than baseline but had improvements at 6 months compared to 3 months. For SBRT patients, the mean sexual QOL declined at 1 month with subsequent return to near baseline sexual QOL by 12 months. At no point was the decline in mean EPIC sexual QOL clinically significant for the SBRT patients.

Mean bowel QOL scores for surgery patients exhibited a small, but clinically significant decline at 1 month followed by recovery to near baseline by 6 months. For SBRT patients, the mean bowel scores exhibited a clinically significant decline at one month that improved by 6 months. The mean bowel scores for SBRT patients remained clinically significantly lower than baseline until 12 months after which they were no longer clinically significantly different from baseline.

Table 3 summarizes the results from the GEE models to assess impact at different follow-up times. For the urinary model, patients receiving SBRT had significantly higher QOL throughout follow-up with the largest difference at one month. For the sexual model comparison of SBRT to all surgery patients, surgery patients had significantly lower sexual QOL at all time points. For the bowel model, the most significant difference occurs at 1 month where surgery patients had significantly higher QOL.

**Table 3:** Generalized estimating equation models of the association between EPIC scores surgery and SBRT patients

**Variable**	**EPIC urinary**			**EPIC sexual**			**EPIC bowel**		
	**β**	**SE**	***p***	**β**	**SE**	***p***	**β**	**SE**	***p***
Intercept	98.9668	4.7493	<.0001	133.2671	9.3670	<.0001	103.1127	2.8919	<.0001
Age	−0.0358	0.0695	0.6062	−1.2686	0.1368	<.0001	−0.0954	0.0435	0.0282
Prostate Volume	−0.0209	0.0227	0.3557	−0.0014	0.0382	0.9702	−0.0158	0.0121	0.1927
Risk Group									
Low	Ref.			Ref.			Ref.		
Intermediate-high	0.7602	1.1711	0.5163	1.2493	2.0884	0.5497	0.6897	0.5534	0.2127
Treatment Group (differences at baseline)									
Surgery	Ref.			Ref.			Ref.		
SBRT	−6.1083	1.1334	<.0001	11.1251	2.6339	<.0001	−0.2676	0.6838	0.6956
Interaction surgery x time (change from baseline)									
Baseline	Ref.			Ref.			Ref.		
1 month	−48.5886	3.3500	<.0001	−38.7584	2.7183	<.0001	−2.4796	1.1901	0.0372
6 months	−19.2903	2.1428	<.0001	−34.6929	2.2146	<.0001	−0.2362	0.8788	0.7881
12 months	−13.3859	1.8891	<.0001	−29.6338	2.2676	<.0001	1.1840	0.7165	0.0984
24 months	−14.1136	1.8888	<.0001	−30.1501	2.5436	<.0001	1.4723	0.6095	0.0157
36 months	−12.8108	1.6371	<.0001	−27.3960	2.4127	<.0001	2.6794	0.5251	<.0001
Interaction SBRT x time (differences from surgery in change from baseline)									
Baseline	Ref.			Ref.			Ref.		
1 month	32.8134	3.3992	<.0001	31.3269	2.8315	<.0001	−18.7600	1.4602	<.0001
6 months	17.8838	2.4335	<.0001	30.6081	2.6451	<.0001	−3.5163	1.4358	0.0143
12 months	11.4988	1.9338	<.0001	26.2387	2.3241	<.0001	−2.6986	1.0348	0.0091
24 months	13.3402	1.9474	<.0001	24.6944	2.6305	<.0001	−1.8731	0.9630	0.0518
36 months	13.6449	1.6969	<.0001	19.8132	2.5509	<.0001	−2.5845	0.6613	<.0001

## Discussion

As the long-term efficacy of SBRT becomes established and other prostate cancer treatment approaches are refined and improved, continued research into QOL after prostate cancer treatment is critical in driving both patient and clinical treatment decisions. Indeed, 5-year follow-up for SBRT has yielded disease control that approximates that produced by surgery and other conventionally fractionated radiation therapy treatments [3]. Thus, preservation of QOL may become the primary basis upon which patients choose a prostate cancer treatment. It is in this context that we present the first study to compare QOL after SBRT and radical prostatectomy, with QOL assessed at approximately the same times pre- and post-treatment and using the same validated QOL instrument (EPIC). In this study, the largest differences in QOL occurred in the first 1–6 months after treatment, demonstrating larger declines following surgery in urinary and sexual QOL as compared to SBRT, and a larger decline in bowel QOL following SBRT as compared to surgery. Overall, these results may have implications for patient and physician clinical decision making which are often influenced by QOL.

Urinary and sexual function are the primary concerns for men undergoing prostate cancer treatment [9,14,16]. Urinary QOL is typically altered by several types of urinary complications. Incontinence, leakage of urine, is the predominant urinary issue after surgery [14,16] with 3 month continence rates of 51-74% for radical prostatectomy [23-25] that improve to 80-94% by 12 months [23,26]. Incontinence is not typically a concern following SBRT [4,5,27,28], more typical are urinary toxicities such as urinary retention, urgency or hesitancy at rates of 2-10% [3-5,27-29]. A major urinary factor in surgery is that the urethra is cut in half during surgical removal of the prostate and then reconnected during the surgery, whereas the urethra remains completely intact and is identified and avoided during SBRT. As a result surgery patients require a catheter that stays in place following discharge from the hospital for 7 to 10 days after treatment and often patients must use pads to address urine leakage in the months following treatment. In this analysis, the overall EPIC urinary domain was analyzed, but the EPIC urinary sub-domain scores for incontinence and obstruction were not analyzed. For the SBRT patients, only the overall urinary summary score for each patient was recorded which prevented analysis of the sub-domain scores in this analysis. A prior examination of this EPIC surgery data using the obstruction and incontinence sub-domains showed urinary obstructive QOL had a transient decline at one month followed by return to baseline with even improvement for patients with preexisting urinary irritative-obstructive symptoms, whereas urinary incontinence QOL significantly impacted surgery patients at all time points [16].

In the current study, SBRT patients had significantly higher sexual QOL after treatment than surgery patients with no clinically significant decline in sexual QOL at any of the study’s follow-up time points. In contrast, the surgery patients, whether or not the nerve-sparing technique was used, had clinically significant declines in sexual QOL at all time points. This is not surprising given the difficulty in identifying the neurovascular bundle in the operating room and the mechanical and thermal injury that may occur to the nerves during surgery. While robotic assisted laparoscopic prostatectomy is more commonly used in the United States, the surgical patients in this study received open radical retropubic prostatectomy, as laparoscopic surgery was not routinely offered in the Spanish hospitals at the time of their study. It remains unknown how significant of an impact laparoscopic (either robotic or not) has on sexual QOL as published functional outcomes vary greatly. For example, potency rates at 12 months for robotic-assisted laparoscopic prostatectomy range from 61–97% [30-34] with patient selection, potency definition, surgeon experience and use of penile rehabilitation primarily accounting for the large variation in rates [33]. Indeed, a recent review by Finkelstein et al. at NYU in which they examine multiple comparative publications states “it is difficult to determine if one approach is superior to the other for the preservation of neurovascular bundles and sexual function” [35].

Baseline mean EPIC sexual QOL scores for the patients receiving SBRT and nerve-sparing surgery in the current study were similar whereas the patients receiving non-nerve-sparing surgery had significantly lower baseline mean EPIC sexual QOL scores. This low baseline sexual QOL likely reflects why these patients were not chosen for the nerve-sparing procedure in that their sexual function was already grossly impaired. Mean EPIC sexual scores were observed to drop dramatically in the short term after surgery independent of surgical approach. While patients receiving nerve-sparing surgery had a greater degree of recovery neither group recovered back to baseline levels. In contrast, mean EPIC scores after SBRT declined slowly over time but were not clinically significantly different than the baseline levels. Failure to return to baseline sexual function is not uncommon following prostatectomy. Levinson et al., found 27% of 568 patients receiving laparoscopic radical prostatectomy returned to baseline EPIC sexual QOL at 24 months [36]. Similarly, while in a study of potent patients Willis et al. observe a greater return to baseline function for patients receiving robotic-assisted laparoscopic prostatectomy the mean EPIC sexual QOL score at 12 months remained 66.2 and 73.7% of the baseline score for robotic-assisted laparoscopic prostatectomy and laparoscopic prostatectomy, respectively [37].

Wiegner and King at Stanford University assessed sexual QOL using EPIC for 32 low-risk prostate cancer patients who received SBRT (36.25 Gy in 5 fractions) [38]. The mean baseline sexual QOL score of 67.5 declined to 56.4, 50.7 and 37.4 at 12, 20 and 50 months, respectively. McBride et al. report sexual QOL using EPIC for 26 patients treated with SBRT (primarily 35 or 36.26 Gy in 5 fractions) as part of a multi-institutional study [29]. The median baseline sexual QOL score of 43 declined to 17 at 36 months. The small number of patients in both of these studies may reflect upon the larger observed declines in sexual QOL. These studies also consisted of patients treated very early in the groups’ SBRT experience and prior to adoption of more advanced and currently used SBRT techniques, such as advanced collimation that have been shown to spare more normal tissue during treatment [39,40]. In addition, the Stanford report did not incorporate the use of MRI which is routinely used to definitively identify the neurovascular bundles in treatment planning.

Mild to moderate bowel toxicity consisting of proctitis and rectal bleeding has been reported following SBRT [4,29,41]. In contrast, very low rates of bowel injury have been reported during prostatectomy [42,43], as bowel complications are not typically an issue for patients undergoing surgery. In the current study, bowel QOL was minimally affected by surgery whereas bowel QOL fell after SBRT but returned to near baseline 6–12 months post-treatment. This is consistent with studies showing the incidence of Grades 2 and 3 bowel toxicity after SBRT (as measured using the RTOG scale) range from 4–24% in the acute phase, but falls to 1-12% in the late phase [4,5,28,29,41,44-46].

The present study is limited by the retrospective nature of the analysis despite the prospective data collection for the two treatment groups. First, treatment groups differed at baseline in ways that might be expected of such a retrospective analysis, and which might have contributed to post-treatment differences between groups. SBRT patients were older, on average, than surgery patients. Although the influence of age was considered by including it in the GEE models, adjusting for age is not sufficient since we do not have data for surgery patients older than 74 years. Differences in the use of treatment for erectile dysfunction may affect sexual outcomes; for example, baseline sexual scores of the older patients in the SBRT group were as good or better than the surgery patients, suggesting a possible relationship with higher sildenafil intake among SBRT patients. However, this could not be included as a baseline adjustment because patients started sildenafil use at various timepoints throughout the study. Other lifestyle factors such as smoking, marital status, and education, and comorbidities were not explicitly balanced between groups so their contribution cannot be assessed. General QOL at baseline was also not assessed. Second, nerve-sparing techniques were not widely applied in the surgery group (28% of patients treated with prostatectomy), and therefore, our findings from the GEE model for the prostatectomy group as a whole could overestimate their adverse sexual effects. Third, both the SBRT and surgery groups had small numbers of patient responses early in their studies. For the surgery patients, the 1-month follow-up had the fewest responses whereas for the SBRT group the 6 month follow-up had the fewest. For the sexual domain, GEE analysis excluding the 1- and 6-month time points showed similar results, suggesting these fluctuations in questionnaire completion did not greatly impact the results. Fourth, there are QOL domains that are not assessed by EPIC which may differ between groups, e.g., fatigue and general quality of life. A more extensive analysis of a broader range of QOL variables may permit more complete comparisons. Some aspects of treatment could not be rigorously controlled, including surgeon expertise which is known to affect disease-control and toxicity outcomes. These issues of heterogeneity across groups could only be addressed effectively in the context of a randomized design. Finally, prostate treatment is an evolving practice; results such as these are based on a limited snapshot of current practices. The emphasis on QOL in prostate cancer patients demands continuing re-analyses of QOL outcomes as treatments evolve.

## Conclusions

This is the first study to examine comparative QOL after SBRT and radical prostatectomy for which differences in QOL occurred immediately following treatment after which relevant, but less substantial differences remained for up to 3 years. These differences in sexual, urinary and bowel QOL should be closely considered in selecting the right treatment for patients and important factors in the overall treatment decision process, especially in evaluating the value of non-invasive treatments, such as SBRT. SBRT had a greater impact on short-term bowel QOL than surgery but had clinically significant advantages in preserving urinary and sexual QOL as compared to surgery. Long-term urinary and sexual QOL declines remained clinically significantly lower for surgery patients but not for SBRT patients. While additional research is needed to understand how different surgical approaches may impact QOL, incontinence and sexual dysfunction remain the primary concern for surgery patients. In particular for low-risk patients who may reasonably be considered for active surveillance, preservation of QOL may become the primary basis for choosing a prostate cancer treatment.

## Abbreviations

EPIC: Expanded prostate cancer index composite; GEE: Generalized estimating equation; GTV: Gross tumor volume; PSA: Prostate-specific antigen; PTV: Planning target volume; SBRT: Stereotactic body radiation therapy; QOL: Quality of life.

## Competing interests

AK has received reimbursement as a consultant for Accuray, Inc. MF and JS have no financial conflicts of interest.

## Authors’ contributions

AK and JS treated the patients. AK and MF performed the quality of life assessments. AK conceived the study and drafted the manuscript. All authors read, edited and approved the final manuscript.

## Authors’ information

**Participants in the Multicentric Spanish Group of Clinically Localized Prostate Cancer**: Jordi Alonso, Adela Virginia Becerra, Oriol Cunillera, Montse Ferrer, Olatz Garín, Yolanda Pardo, Angels Pont (IMIM- Hospital del Mar Research Institute); Ana Boladeras, Ferran Ferrer, Ferran Guedea, Evelyn Martínez. Victoria Eugenia Padin, Joan Pera, Montse Ventura (Institut Català d’Oncologia); Ferran Aguiló, José Francisco Suárez (Hospital Universitari de Bellvitge); Sergio Pastor, Josep Maria Prats (Corporació de Salut Maresme i la Selva); Javier Ponce de León, Humberto Villavicencio (Fundación Puigvert); Jose Emilio Batista (Fundación Teknon); Jordi Craven-Bratle, Gemma Sancho (Hospital de la Santa Creu i Sant Pau); Adriana Ayala, Belen de Paula, Pablo Fernández (Instituto Oncológico de Guipúzcoa); Benjamin Guix (Fundación IMOR); Ismael Herruzo (Hospital Regional Carlos Haya); Helena Hernández, Víctor Muñoz (Hospital Meixoeiro- Complejo CHUVI); Asunción Hervas, Alfredo Ramos (Hospital Ramon y Cajal); Víctor Macias (Hospital Cínico Universitario de Salamaca); Josep Solé, Marta Bonet (Institut Oncologic del Valles -IOV); Pilar Marcos (Capio Hospital General de Catalunya); Alfonso Mariño (Centro Oncológico de Galicia); María José Ortiz (Hospital Virgen del Rocío); Pedro J. Prada (Hospital Universitario Central de Asturias).
